# Comparison of Thyroid Blood Flow and Uptake Indices Using Technetium-99m Pertechnetate in Patients with Graves’ Disease and Euthyroid Subjects

**DOI:** 10.4274/mirt.58076

**Published:** 2014-10-05

**Authors:** Hamid Javadi, Ali Mahmoud Pashazadeh, Mehdi Mogharrabi, Iraj Nabipour, Mohammadreza Kalantarhormozi, Majid Assadi

**Affiliations:** 1 Golestan University of Medical Sciences, Departement of Nuclear Medicine, Golestan Research Center of Gastroenterology and Hepatology (GRCGH), Gorgan, Iran; 2 Bushehr University of Medical Sciences, The Persian Gulf Nuclear Medicine Research Center, Bushehr, Iran; 3 Bushehr University of Medical Sciences, The Persian Gulf Tropical Medicine Research Center, Department of Endocrinology, Bushehr, Iran

**Keywords:** Regional blood flow, Radionuclide imaging, perfusion imaging, Graves’ disease

## Abstract

**Objective:** The aim of the present study was to evaluate parameters of early blood flow (EBF) including duration of EBF, perfusion index (PI), uptake indexes 1 and 2 (UI1 and UI2) derived from dynamic thyroid scintigraphy in patients with Graves’ disease (GD), and euthyroid (EI). Maximum instant uptake and maximum uptake of the thyroid gland were also assessed.

**Methods:** A total of 50 patients with GD and EI were included in this study. Each patient underwent two dynamic scans of 1-minute (20 images at 3 seconds) and 20-minute (20 images at 1 minute) with ^99m^TcO4. The time-activity curve of a 1-minute scan was employed to derive EBF parameters; likewise, the time-activity curve of a 20-minute scan was used to determine maximum instant uptake, and the gradient of the curve from the 10th to 20th minute was used to assess maximum uptake.

**Results:** Values of EBF duration, PI, UI1, UI2, and maximum instant uptake were significantly lower in patients with GD than in those with EI (p<0.05). The calculated gradient of the second half of the curves for all of the patients ranged from 0 to 0.88 and was significantly higher in patients with GD than in those with EI.

**Conclusion:** Lower values of PI, UI1, UI2 and durations of EBF, along with faster maximum instant uptake in patients with GD in comparison to EI are indicators of a heightened desire for hyper-functioning thyroid glands of patients with GD to absorb ^99m^TcO4. Additionally, because of the uprising gradient at the end of the 20-minute time-activity curve, a maximum thyroid uptake of ^99m^TcO4 was achieved at more than 20 minutes after the radiopharmaceutical injection.

## Abstract (tr)

**Amaç:** Bu çalışmanın amacı Graves’ hastalığı (GH) olan hastalarda ve ötiroid (Ö) kişilerde yapılan dinamik tiroid sintigrafisinden elde edilen en kan akımı (EKA) süresi, perfüzyon indeksi (PI), uptake indeksi 1 ve 2 (UI1 ve UI2) gibi erken kan akımı parametrelerinin değerlendirilmesi idi. Tiroid bezinin maksimum uptake ve maksimum anlık uptake de araştırıldı.

**Yöntem:** GH ve Ö olan toplam 50 hasta çalışmaya dahil edildi. Her hastaya ^99m^TcO4 ile 1 dakikalık (3 saniyelik 20 görüntü) ve 20 dakikalık (1 dakikalık 20 görüntü) iki dinamik görüntüleme yapıldı. Bir dakikalık görüntülemenin zaman aktivite eğrisi EKA parametrelerini, 20 dakikalık görüntülemenin zaman aktivite eğrisi de maksimum anlık uptake değerini, 10-20 dakika arasındaki eğrinin eğimi ise maksimum uptake değerini hesaplamada kullanıldı.

**Bulgular:** EKA süresi, PI, UI1, UI2 ve maksimum anlık uptake değerleri, GH olan hastalarda Ö hastalara göre anlamlı olarak daha düşüktü (p<0,05). Eğrinin ikinci yarısının hesaplanan eğimi tüm hastalarda 0 ile 0,88 arasında değişiyordu ve GH olan hastalarda Ö hastalara göre anlamlı olarak daha yüksekti.

**Sonuç:** Ö hastalarla karşılaştırıldığında, GH hastalarında PI, UI1, UI2 ve EKA süresinin düşüklüğü hızlı maksimum anlık uptake ile birlikte, hiperfonksiyone tiroid bezinin ^99m^TcO4’ü absorbe etmekteki artmış isteğini gösterir. Ayrıca 20 dakikalık zaman aktivite eğrisinin sonundaki artmış eğim sebebiyle, ^99m^TcO4’ün maksimum tiroid uptake’ine radyofarmasötik enjeksiyonundan 20 dakikadan daha sonra ulaşılmaktadır.

## INTRODUCTION

Various types of thyroid disorders may affect normal function of the thyroid glands. For instance, in Graves’ disease, thyroid stimulating hormone receptor antibodies (TSHR-Ab) bind thyroid stimulating hormone receptor (TSHR) and activate it, leading to hyper-secretion, hypertrophy, and hyperplasia of the thyroid follicles (1). One of the features of this interruption may appear in thyroid blood flow (2,3,4,5,6). Therefore, evaluation of this parameter may be an indicator in assessment of thyroid gland function.

A number of methods have been introduced and employed to assess thyroid blood flow including angiography, electromagnetic flowmetry, doppler ultrasonography, and nuclear medicine imaging. Because of problems associated with the invasiveness of angiography and electromagnetic flowmetry, these methods are not commonly utilized; however, application of ultrasonography for this purpose benefits from simplicity, noninvasiveness, and the potential for quick differentiation between diffuse and nodular thyroid disease (7,8,9,10). Application of diagnostic nuclear medicine techniques for this purpose-thyroid scintigraphy-is associated with favorable sensitivity and specificity which provides images of the vascularity of “cold” thyroid nodules, which can be used to distinguish neoplastic from non-neoplastic ones (8,11,12,13). Because of the similarity of the ^99m^TcO4 with iodine (with respect to the molecular size), thyroid uptake of ^99m^Tc can be used to assess thyroid function (14).

It is accepted that the maximum thyroid uptake from ^99m^TcO4 is usually achieved at 10 to 20 minutes after intravenous injection of the radiotracer. As maximum thyroid uptake of the radiopharmaceutical is necessary to the proper evaluation of thyroid function, the 20-minute thyroid uptake is commonly used to estimate thyroid function (15,16,17).

The aim of the present study was to assess and compare parameters of EBF derived from dynamic thyroid scintigraphy in patients with GD and EI. Also, maximum instant uptake of ^99m^Tc uptake over 20 minutes was determined and compared for all patients with GD and EI. We assessed whether scintigraphy of the thyroid at 20 minutes after intravenous injection of ^99m^Tc provides the required time for maximum uptake of radiopharmaceuticals.

## MATERIALS AND METHODS

A total of 50 patients with GD (37) and EI (13) were included in this prospective study. Diagnosis of the disease was based on clinical and laboratory findings. In their documents, patients with GD experienced symptoms such as hyperthyroidism and elevated levels of thyroid hormones. In the documents of the EI, no symptoms of hyperthyroidism were observed and levels of TT3, TT4, and TSH were normal.

Following the intravenous injection 5 mCi ^99m^TcO4 as a bolus, thyroid scintigraphy of each patient was performed in the supine position with neck extended. A gamma camera (ADAC Pegasys; model SH Genesys Epic) was positioned 7 cm over the thyroid gland. For each patient, dynamic scintigraphy was performed in two steps: first, in order to study perfusion of the thyroid gland, each patient was scanned over 1 minute as 20 frames of 3 seconds; second, in order to assess thyroid uptake, each patient was scanned over 20 minutes as 20 frames of 1 minute. Results of the scans were recorded on a 64×64 matrix using a zoom factor of 1.5.

Time-activity curves of the patients were generated following the background subtraction. In order to do this, one region of interest (ROI) was drawn around the thyroid gland to be used to determine thyroid activity while another ROI was drawn below the gland to be used to determine background activity.

**Figure 1:**
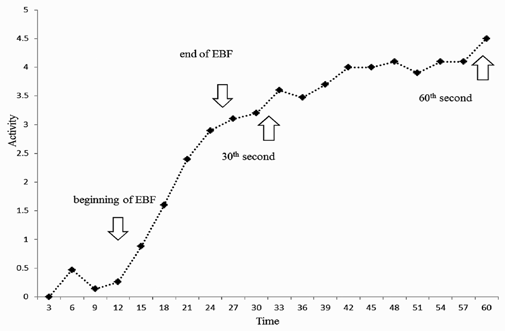
Typical 1-minute time-activity curve (time in seconds)

Parameters of thyroid blood flow were derived from time-activity curves similar to the method applied by Sekulic et al. (14) as follows:

EBF duration, which is the interval time between the start and the end of EBF curve; the perfusion index, which is the ratio of counts at the beginning of the EBF to the counts at the end of the EBF; the uptake index 1, which is the ratio of the counts at the end of EBF to the counts at the 60th seconds; and the uptake index 2, which is the ratio of the counts at the 30th seconds to the counts at the 60th seconds (Figure 1).

**Figure 2:**
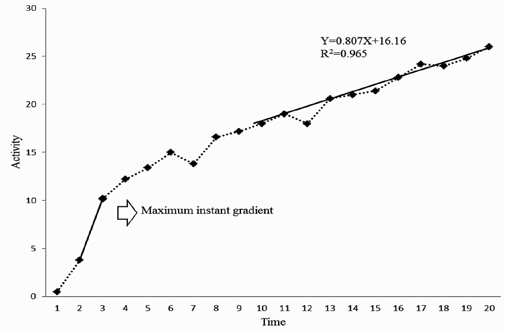
Typical 20-minute time activity curve (time in minutes)

In order to assess if scintigraphy of the thyroid at the 20th minute after intravenous injection of ^99m^TcO4 provides the required time for maximum uptake of the radiopharmaceutical, in a time-activity curve, the gradient of the line fitted to the second 10 minutes of the curve (from 10th to 20th minutes) was determined. Also, the time point during the 20-minute scans in which the highest amount of ^99m^TcO4 uptake would occur (maximum instant uptake) was determined for all patients with GD and EI (Figure 2).

**Statistical Analysis**

In each case, the curves were acquired utilizing the Analysis ToolPak added to Microsoft Excel. All obtained data were provided with mean ± SE, minimum, and maximum values. Comparison of data between GD and EI was performed using a Mann-Whitney U test. A p value of less than 0.05 was considered statistically significant. SPSS for Windows software package (Release 18, SPSS Inc., Chicago, Illinois) was used for the statistical analysis.

## RESULTS

All of the patients with GD and EI underwent dynamic thyroid scintigraphy with ^99m^TcO4. After the background subtraction, recorded activities were used to generate corresponding time-activity curves of the 1-minute and 20-minute scans.

The 1-minute time-activity curves were used to extract semi-quantitative parameters of thyroid uptake of ^99m^TcO4. A summary of the resulting data is presented in Table 1. As shown in the table, duration of EBF in the 1-minute time-activity curve was significantly lower in patients with GD in comparison to those with EI (p<0.05). It was also observed that UI1 and UI2 in patients with GD were significantly faster than in EI (p<0.05). The difference in PI between patients with GD and EI was also significant (p<0.05).

In the 20-minute time-activity curves, maximum instant gradient during 20 minutes for patients with GD and EI were determined as 4.30 and 5.54 minutes, respectively. According to the corresponding data, the time required for the thyroid to reach maximum instant gradient in patients with GD was significantly shorter than in those with EI (p<0.05).

The gradient of the best-fit line to the curve from the 10th to 20th minute was calculated respectively as 0.125 and 0.0043 for the patients with GD and EI, indicating a statistically significant difference between the two groups (p<0.05) (Table 2).

## DISCUSSION

In previous studies, it was found by ultrasonography that thyroid vascularity-and therefore thyroid blood flow-increased in patients with hyperthyroidism (5). In addition, TSH is able to stimulate the expression of growth factors in thyroid follicular cells, which in turn results in the synthesis of endothelium. One of the consequences observed following the synthesis of endothelium is blood capillary enlargement and a resulting increase in thyroid blood flow. In patients with GD, a similar process may occur with TSHR-Ab (18). Therefore, further study of the thyroid blood flow may provide vital information for these patients.

Evaluation of the vascularity of thyroid glands with ^99m^TcO4 scintigraphy has been reported in many studies (8,12,19,20). As a consequence of vascularity, concentration of radioactivity has been increased in the thyroid gland of patients with GD.

In the present study, results of 1-minute dynamic scans which were performed to semi-quantitatively assess parameters of thyroid blood flow and uptake of ^99m^TcO4, revealed that duration of early blood flow in patients with GD is significantly shorter than in those with EI (p<0.05). It was also found that uptake index 1 and 2 in patients with GD is faster than in those with EI, and the observed difference was statistically significant (p<0.05). These findings related to the increased vascularity and hyper-functioning thyroid glands in patients with GD were consistent with the results of a similar study performed by Sekulic et al. (14). However, in our study the perfusion index (PI) was smaller in patients with GD than in those with EI; therefore our study was not completely concordant with the results of the study performed by Sekulic et al. may be due to small numbers in both studies and also different included population in each study which may be due to small sample size and also different included population in each study.

As indicated in the literature and guidelines, thyroid glands achieve maximum uptake 20 minutes after the injection of ^99m^TcO4. For this reason, thyroid static scans are routinely performed following this period of time to estimate thyroid function. In this study, 20-minute dynamic scans of the thyroid uptake provided an opportunity to assess maximum thyroid uptake semi-quantitatively. Analysis of the equation of the best line fitted to the second half (from 10th to 20th minutes) of each generated time-activity curve indicated that, in the majority of patients with GD and EI, gradients of these lines were not zero and extended up to 0.88. The greater the gradient of the fitted line to the time-activity curve, the greater the tendency of the thyroid gland to reach its maximum uptake after 20 minutes. On the other hand, this semi-quantitative analysis indicated that static scintigraphy of the thyroid gland 20 minutes after the injection of ^99m^TcO4 might not accurately estimate thyroid function, and that a longer time interval might provide more reliable information. Comparison of the mean values of these gradients in patients with GD and EI revealed that this quantity is significantly higher in patients with GD than in those with EI. This means that patients with GD require much longer times than those with EI in order for maximum uptake to be achieved.

Based on analysis of the gradients of the time-activity curve in each of 20 scans taken over 20 minutes (20 gradients for each curve), we also found that maximum instant uptake (among the 20 gradients for each curve) of thyroid gland occurs more quickly in patients with GD than in those with EI. The reason for our findings regarding maximum uptake and maximum instant uptake of thyroid might be related to the function of thyroid glands in patients with GD, in which the malfunctioned gland has more desire to absorb and keep ^99m^TcO4 in the earlier moments after the injection of the radiopharmaceutical and tends to absorb it for a longer time.

## CONCLUSION

Thyroid glands of patients with GD, in comparison with those found in patients with EI, demonstrate faster UI1 and UI2, and maximum instant uptake occurs faster. In order to obtain more reliable statistical scans of thyroid glands with ^99m^TcO4, we recommend that a duration longer than 20 minutes be considered between injection of the radiopharmaceutical and performance of the scintigraphy. However, further studies investigating dynamic scans of the thyroid gland at intervals greater than 20 minutes are recommended to support such an assumption.

**Conflicts of Interest**

There are no conflicts of interest.

## Figures and Tables

**Table 1:**
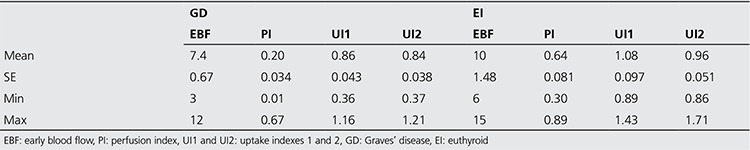
Duration of EBF (in seconds), Perfusion Index, and Uptake Index 1 and 2 based on the 1-minute time activitycurve in patients with GD and EI

**Table 2:**
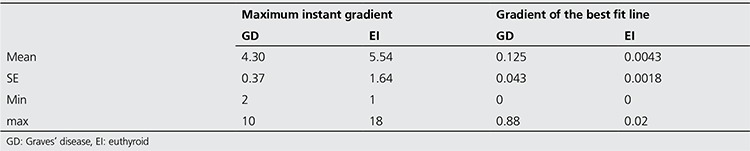
Corresponding time (in minutes) to the maximum gradient and gradient of the best fit line to the curve (from10th to 20th minute) in the 20-minute time activity curve
